# A co-evolutionary arms race: trypanosomes shaping the human genome, humans shaping the trypanosome genome

**DOI:** 10.1017/S0031182014000602

**Published:** 2014-06-26

**Authors:** PAUL CAPEWELL, ANNELI COOPER, CAROLINE CLUCAS, WILLIAM WEIR, ANNETTE MACLEOD

**Affiliations:** Wellcome Trust Centre for Molecular Parasitology, College of Medical, Veterinary and Life Sciences, University of Glasgow, 464 Bearsden Road, Glasgow G61 1QH, UK

**Keywords:** Co-evolution, Genome, Trypanosome

## Abstract

*Trypanosoma brucei* is the causative agent of African sleeping sickness in humans and one of several pathogens that cause the related veterinary disease Nagana. A complex co-evolution has occurred between these parasites and primates that led to the emergence of trypanosome-specific defences and counter-measures. The first line of defence in humans and several other *catarrhine* primates is the trypanolytic protein apolipoprotein-L1 (APOL1) found within two serum protein complexes, trypanosome lytic factor 1 and 2 (TLF-1 and TLF-2). Two sub-species of *T. brucei* have evolved specific mechanisms to overcome this innate resistance, *Trypanosoma brucei gambiense* and *Trypanosoma brucei rhodesiense*. In *T. b. rhodesiense*, the presence of the serum resistance associated (*SRA*) gene, a truncated variable surface glycoprotein (VSG), is sufficient to confer resistance to lysis. The resistance mechanism of *T. b. gambiense* is more complex, involving multiple components: reduction in binding affinity of a receptor for TLF, increased cysteine protease activity and the presence of the truncated VSG, *T. b. gambiense*-specific glycoprotein (*TgsGP*). In a striking example of co-evolution, evidence is emerging that primates are responding to challenge by *T. b. gambiense* and *T. b. rhodesiense*, with several populations of humans and primates displaying resistance to infection by these two sub-species.

## INTRODUCTION

*Trypanosoma brucei* is the causative agent of African sleeping sickness in humans and one of several species that causes the related veterinary disease Nagana. Both diseases have a wide distribution across sub-Saharan Africa and affect some of the poorest areas of the world. *Trypanosoma brucei* is traditionally segregated into three morphologically identical sub-species based on host, geography and pathology, although the polyphyletic nature of the three sub-species and evidence for mating between them makes grouping them as such problematic. *Trypanosoma brucei brucei* is limited to domestic and wild animals throughout sub-Saharan Africa and is non-infective to humans (and some primates) due to sensitivity to trypanosome lytic factors (TLF) found in its serum (Seed and Sechelski, 1990; Lugli *et al.* 2004). *Trypanosoma brucei gambiense* and *Trypanosoma brucei rhodesiense* are human infective sub-species, named due to their relative geographic locations. *Trypanosoma b. gambiense* is found in Western and Central sub-Saharan African and causes a chronic infection that can persist for many years before symptoms appear (Gibson, 1986). It appears to be largely a disease limited to humans, although some animal reservoirs have been described (Gibson *et al.* 1978; Mehlitz, 1979; Felgner *et al.* 1981; Mehlitz *et al.* 1981, 1982; Zillmann *et al.* 1984). *Trypanosoma b. rhodesiense* is found in a limited but expanding area of Eastern sub-Saharan Africa, and there is a large potential animal reservoir in wild and domesticated animals (Welburn *et al.* 2001; Fèvre *et al*. 2001, 2005; Odiit *et al.* 2005). This sub-species causes a more acute form of the disease than *T. b. gambiense* (Gibson, 2002), although *T. b. gambiense* is by far the more prevalent human-infective sub-species and is responsible for more than 97% of human cases (World Health Organization, 2006; Simarro *et al.* 2011). The evolution of TLF in primates and the adaptations of *T. brucei* sub-species to resist lysis represent an excellent example of the co-evolutionary arms race between host and parasite.

## HUMAN RESISTANCE TO TRYPANOSOME INFECTION

**Fig. 1.**
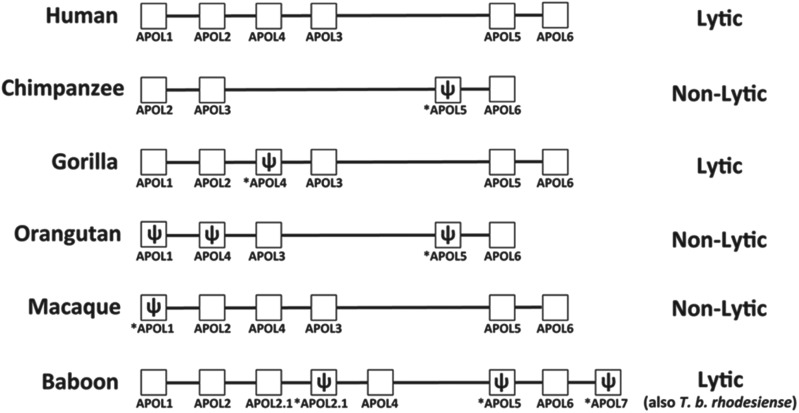
APOL gene cluster of various primate species. The complement of APOL genes and pseudogenes in several primate genera. The proposed genomic arrangement for each genus is shown, with pseudogenes indicated by *ψ* and *. The potential for the species’ serum to lyse *T. brucei* is also indicated (adapted from Smith and Malik, 2009).

It has been known for more than a century that a component found in the serum of several primates, including humans, is toxic to trypanosomes (Fig. 1) (Laveran and Mesnil, 1912). After exposure to human serum, most trypanosomes are rapidly lysed with a defined morphology (Pays *et al.* 2006). Lysis is preceded by a characteristic swelling of the lysosome, suggesting that this organelle is intimately involved in the process. Fractionation of human serum identified a specific high-density lipoprotein (HDL) particle approximately 500 kDa in size that was able to lyse trypanosomes (Rifkin, 1978*a*, *b*; Hajduk *et al.* 1989). This particle was originally termed the TLF but later renamed TLF-1 after the discovery of a second particle, a high molecular weight serum protein-binding complex related to TLF-1 that was consequently named TLF-2 (Raper *et al.* 1996). Both TLF-1 and TLF-2 contain the same protein complement: apolipoprotein A1 (APOA1), apolipoprotein L1 (APOL1) and haptoglobin-related protein (HPR), although TLF-1 is predominantly composed of lipid while TLF-2 is lipid poor (Raper *et al.* 2001). Despite this, TLF-2 is a much larger particle than TLF-1 due to being bound to several IgM molecules (Tomlinson *et al.* 1995; Raper *et al.* 1996). Considerable effort and much debate has focused on determining the trypanolytic component of TLF, largely focusing on TLF-1 as a result of the difficulty of purifying active TLF-2 *ex vivo* (Tomlinson *et al.* 1995; Raper *et al.* 1996). Investigation has centred on the two primate-specific proteins found in TLF, i.e. HPR and APOL1, both of which are the result of tandem gene duplication events during primate evolution. HPR is a haemoglobin binding protein with high sequence similarity to the haem scavenger protein haptoglobin (HP). Found within the same HDL complex as HPR, APOL1 is a lipid-binding protein that possesses both a secretory signal peptide and a Bcl-2 homology 3 (BH3) domain (Duchateau *et al.* 1997; Wan *et al.* 2008; Zhaorigetu *et al.* 2008). The BH3 domain is an important pro-apoptotic regulator of autophagy in many cell types (Lutz, 2000) and APOL1 itself appears to be able to induce autophagy if over-expressed in cell lines, indicating it may have a role in vertebrate controlled cell death (Zhaorigetu *et al.* 2008). APOL1 mutations are also associated with various pathologies, including schizophrenia and chronic kidney diseases (Mimmack *et al.* 2002; Genovese *et al.* 2010; Tzur *et al.* 2010).

While there has been some controversy concerning the roles of these two proteins with regards trypanosome lysis, the current consensus is that both proteins are necessary for optimal lysis and that HPR and APOL1 have complementary roles (Vanhollebeke *et al.* 2007; Widener *et al.* 2007). Although it has also been shown to have some trypanosome-specific toxicity itself (Harrington *et al.* 2010), possibly due to the un-cleaved signal peptide, which can affect membrane fluidity (Harrington *et al.* 2010), HPR acts primarily as a ligand. Similar to its haptoglobin ancestor, HPR binds haemoglobin and thus facilitates uptake of TLF-1 via a parasite haptoglobin-haemoglobin receptor (HpHbR) (Vanhollebeke *et al.* 2008; Vanhollebeke and Pays, 2010). This strategy likely evolved to take advantage of the fact that trypanosomes are haem auxotrophs (Korený *et al.* 2010). After being endocytosed (Hager *et al.* 1994), the TLF-1 particle is trafficked to the lysosome where APOL1 is released. The hairpin structure of APOL1 is normally configured closed at neutral pH, however, the low pH of the lysosome disperses the salt bridges which stabilize the hairpin ‘hinge’, resulting in a conformational change (Vanhollebeke and Pays, 2006). This reveals a membrane-addressing domain and the BH3 pore-forming domain (Vanhollebeke and Pays, 2006). With both of these domains exposed, APOL1 embeds in the lysosomal membrane, forming anionic pores and thus perturbing the osmotic balance of the organelle. This leads to lysis of the parasite, either by simple mechanical disruption due to swelling of the organelle or the leakage of digestive enzymes into the main cell body (Vanhamme *et al.* 2003; Pays *et al.* 2006; Vanhollebeke and Pays, 2010). As both HPR and APOL1 are found in TLF-2, it is likely that the lysis method for this particle involves both of these proteins, although the method of entry for TLF-2 only partially involves the HpHbR receptor (Bullard *et al.* 2012). The mechanism by which the majority of TLF-2 is bound and internalized by the parasite is currently unknown, but may involve weak interactions between IgM and the VSG coat of the parasite or a second receptor for TLF that has been described (Drain *et al.* 2001; Green *et al.* 2002; Vanhollebeke and Pays, 2010). The second trypanosome receptor for TLF is unidentified but was first described from low temperature binding assays showing TLF-1 is internalized by both a high-affinity low copy number receptor (now known to be HpHbR) and a second low-affinity, high copy number receptor (Drain *et al.* 2001). The putative second receptor was further investigated using HDL competition, binding and uptake assays (Green *et al.* 2002) and shown to act as a general lipoprotein scavenger which binds multiple classes of lipoprotein, including HDL, LDL, oxidized LDL and TLF.

Primates have likely evolved this highly effective innate immunity based on APOL1 because trypanosomes have escaped the effects of the adaptive immune response due to antigenic variation (Barry and McCulloch, 2001; Pays *et al.* 2001). Trypanosomes are covered in a highly immunogenic, dense, variable surface glycoprotein coat, which is rapidly turned over with the parasite membrane and frequently switched to a different VSG, rendering antibody-based immunity ineffective. The high turnover of the parasite's surface membrane requires a large amount of lipid, however, trypanosomes are lipid auxotrophs and so obtain their lipids in the form of HDL from their host (Green *et al.* 2002). The packaging of APOL1 into HDL exploits the parasite's requirement for this essential nutrient to deliver the highly toxic APOL1 molecule.

Dating the emergence of trypanolytic APOL1 in primates is challenging as the APOL gene cluster has undergone numerous expansion, duplication and loss events during primate evolution (Fig. 1) (Smith and Malik, 2009), although it must precede the time when the *hominidae* and *cercopithecidae* primate lineages diverged as both possess lytic APOL1 (approximately 20 mya) (Seed and Sechelski, 1990; Lugli *et al.* 2004). The addition of a secretory signal peptide to APOL1 appears to be a unique key factor allowing its use as a protective element against trypanosomes, as all other APOL proteins are intracellular (Vanhollebeke and Pays, 2006). The signal peptide appears to have originated due to a gain of function mutation after the divergence of APOL1 and APOL2 (Smith and Malik, 2009). Although the APOL1 defensive strategy works successfully against most species of African trypanosome, two human infective trypanosome sub-species have evolved to counter the innate defence provided by primate APOL1. Interestingly, both sub-species utilize a truncated VSG as an essential component of their resistance mechanisms. As several other genes also derive from VSGs, including the transferrin receptor ESAG6/ESAG7, it would appear that the huge VSG repertoire of these parasites provides a powerful resource that can be utilized by trypanosomes to quickly evolve to infect new hosts and overcome novel challenges (Jackson *et al.* 2013).

## *TRYPANOSOMA B. RHODESIENSE* RESISTANCE TO TLF AND APOL1

**Fig. 2.**
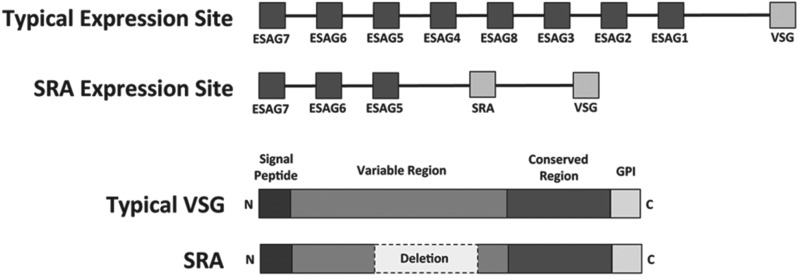
Human serum resistance of *T. b. rhodesiense.* (Upper) Schematic diagram of a typical trypanosome expression site and the SRA expression of *T. b. rhodesiense* (adapted from Gibson, 2005). (Lower) Diagram of the typical domains of a *VSG* and the approximate location of the 378 bp deletion in the *SRA* gene (adapted from Campillo and Carrington, 2003).

Work to elucidate how the human infective sub-species of *T. brucei* overcame APOL1-mediated lysis initially focused on *T. b. rhodesiense*. A defining characteristic of human serum resistance within this sub-species is its variable phenotype. The serum resistance phenotype can vary during animal passage, with individual passages exhibiting the phenotype to differing degrees (Targett and Wilson, 1973; Willett and Fairbairn, 1955). It was noted that the expression of the resistance phenotype closely correlates with changes to the expressed VSG (van Meirvenne *et al.* 1976). This implicated an expression site associated gene (*ESAG)* being responsible for resistance (Fig. 2) (Rifkin *et al.* 1994). Comparison of isogenic serum-susceptible and serum-resistant *T. b. rhodesiense* lines provided an invaluable research tool and by comparing mRNA, a *VSG*-like gene transcript associated with resistance was identified (De Greef *et al.* 1989; De Greef *et al*. 1992). Both resistant and sensitive *T. b. rhodesiense* possess the serum resistance associated (SRA) gene, but only resistant lines actively transcribe it. Conclusive proof that human serum resistance in *T. b. rhodesiense* was due to this single gene was shown when transgenic *T. b. brucei* expressing *SRA* gained resistance to lysis by human serum (Xong *et al.* 1998). Anecdotally, this result was solidified by accidental infection of a human with this *T. b. brucei*–*SRA* strain (Gibson, 2005), demonstrating that the *SRA* gene alone is sufficient to confer human serum resistance.

*SRA* has been shown to be present in nearly all *T. b. rhodesiense* lines (Gibson *et al.* 2002) although this is a circular argument, i.e. the presence of *SRA* is diagnostic of *T. b. rhodesiense*, so *T. b. rhodesiense* must always possess *SRA*. Structurally, the *SRA* gene appears to be a truncated *VSG* with a large deletion of a region in the centre of the sequence encoding the N-terminal domain (De Greef *et al.* 1992; De Greef and Hamers, 1994; Xong *et al.* 1998). SRA is present on the cell surface (Milner and Hajduk, 1999), where it is internalized in the flagellar pocket during cell surface protein turnover, leading to SRA presence in the endosomes and lysosome (Vanhamme *et al.* 2003; Oli *et al.* 2006; Shiflett *et al.* 2007). This is similar to the location of APOL1 in the endosomal pathway before lysis. The deletion of 126 amino acids in SRA removes two surface loops normally present in a VSG protein, exposing the internal *α*-helices (Fig. 2) (Campillo and Carrington, 2003). Based on modelled tertiary structure, it was proposed that the deletion resulted in human serum resistance by allowing an inter-chelating interaction between the exposed *N*-terminal helices of SRA and the *C*-terminal helical section of APOL1 after the protein undergoes conformational change in the acidic environment of the lysosome (Vanhamme *et al.* 2003; Vanhamme and Pays, 2004).

*SRA* is conserved at the nucleotide level in the *T. b. rhodesiense* population, with less than 3% sequence variation between strains (Gibson *et al.* 2002). The polymorphisms present in the *SRA* gene, which can be differentiated by allele-specific PCR (Gibson *et al.* 2002), divide *T. b. rhodesiense* into two groups, ‘Northern’ and ‘Southern’, broadly along geographical lines (Gibson *et al.* 2002; MacLean *et al.* 2004, Balmer *et al.* 2001). This geographical segregation of *SRA* variants associates closely with the molecular and clinical disease profiles that have also detected delineation between Northern and Southern *T. b. rhodesiense* strains (Gibson *et al.* 1980; Hide *et al.* 1991; MacLeod *et al.* 2000; MacLean *et al.* 2004).

The conservation of the *SRA* gene sequence combined with population analyses, which have highlighted the close genetic relationship between sympatric *T. b. rhodesiense* and *T. b. brucei* strains (Hide *et al.* 1994; MacLeod *et al.* 2000), indicate that although the formation of *SRA* is likely to have been the result of a single gene recombination event (Campillo and Carrington, 2003), human infectivity has spread in East Africa by genetic exchange of the *SRA* gene into genetically diverse *T. b. brucei* backgrounds, thereby creating new strains of *T. b. rhodesiense* (Gibson *et al.* 2002; Duffy *et al.* 2013). This potential for genetic exchange between *T. b. brucei* and *T. b. rhodesiense*, alongside the demonstration that expression of the *SRA* gene is all that is necessary for human serum resistance (Xong *et al.* 1998) has implications for the evolution of the disease, as the *SRA* gene may be transferred onto new genetic backgrounds resulting in genotypes with altered pathogenicity.

The discovery of *SRA* has led to advancements in diagnosis and suggested preventative action to combat sleeping sickness caused by *T. b. rhodesiense*. With the advent of specific PCR markers for *SRA*, trypanosomes with the potential to infect humans can be identified unambiguously without the need for laborious human serum resistance assays such as the BIIT (Welburn *et al.* 2001; Gibson *et al.* 2002; Radwanska *et al.* 2002). By providing a robust and specific single gene assay, requiring as little as a single parasite in starting material, SRA-PCR has proved revolutionary in understanding the role of wildlife and livestock in the epidemiology of the disease. For example, traditional microscopy techniques and HSR assays estimated the number of domestic cattle carrying human infective *T. b. rhodesiense* to be approximately 1%. PCR analysis has indicated that instead this value is closer to 18% (Welburn *et al.* 2001). Although the discovery of *SRA* has been a major boon to the understanding of *T. b. rhodesiense* genetics and biology, this gene is not present in the dominant human infective trypanosome sub-species, *T. b. gambiense*. Additionally, several strains have been described from *T. b. rhodesiense* foci that are human serum resistant but for which the *SRA* gene cannot be amplified by PCR (De Greef *et al*. 1992; Enyaru *et al.* 2006). Whether this is due to divergent *SRA* sequence in these strains or a novel resistance mechanism is unknown.

## *TRYPANOSOMA B. GAMBIENSE* RESISTANCE TO TLF AND APOL1

Investigating the human serum resistance phenotype of *T. b. gambiense* has been hampered by the difficulty in working with the sub-species in a laboratory setting. *Trypanosoma b. gambiense* typically grows to very low parasitaemia and there have been immense difficulties in adapting it to *in vitro* or *in vivo* models. However, recent advances have allowed the generation of laboratory adapted lines and successful transfections have now been achieved (Baltz *et al.* 2009; Capewell *et al.* 2013; Uzureau *et al.* 2013). It is now possible to unravel the intricacies of the resistance phenotype of *T. b. gambiense*. An important first step was the observation that *T. b. gambiense* does not appear to internalize fluorescently tagged TLF-1 (Kieft *et al.* 2010; Capewell *et al.* 2011). This suggested that avoidance of TLF-1 is a feature of human serum resistance in *T. b. gambiense*. Several polymorphisms were identified in the *HpHbR* gene unique to *T. b. gambiense* relative to other *T. brucei* that affected TLF-1 uptake (Kieft *et al.* 2010). Subsequent research has confirmed that a single polymorphism reduced the binding affinity of HpHbR for its ligand 20-fold (DeJesus *et al.* 2013; Higgins *et al.* 2013). This polymorphism appears to be conserved across several *T. b. gambiense* foci (Symula *et al.* 2012). Avoidance of TLF-1 therefore appears to be a conserved trait in *T. b. gambiense.* Over-expression of a functional *T. b. brucei* HpHbR in *T. b. gambiense* results in normal TLF-1 uptake and restricted growth (Capewell *et al.* 2013). Incubating wild-type *T. b. gambiense* with serum containing high levels of TLF-1 also appears to result in the serum resistance mechanism of *T. b. gambiense* becoming overwhelmed (Uzureau *et al.* 2013). While the reduced expression and activity of HpHbR provide a plausible explanation for how *T. b. gambiense* avoids lysis by TLF-1, it does not explain how *T. b. gambiense* is able to avoid uptake and consequent lysis by TLF-2. This lytic particle is only partially internalized via HpHbR (Bullard *et al.* 2012). Additionally, *T. b. gambiense* exposed to recombinant APOL1 internalized by non-specific fluid phase endocytosis are still resistant despite observable uptake and trafficking of the lytic protein to the lysosome (Capewell *et al.* 2011).

**Fig. 3.**
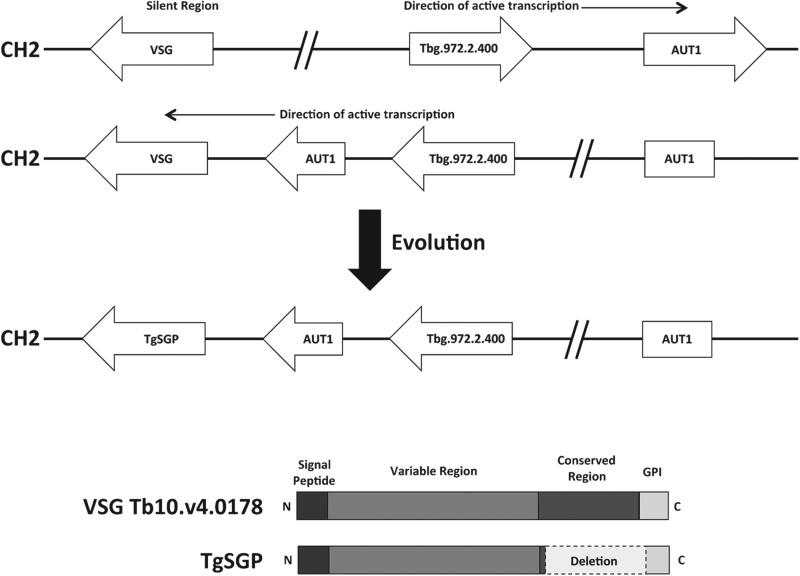
Human serum resistance of *T. b. gambiense.* (Upper) Model of the chromosomal inversion on chromosome 2 that allowed the silent region containing the progenitor of *TgsGP* to be constitutively transcribed and undergo selection. The inversion is present on one homologue of chromosome 2 and is conserved across all *T. b. gambiense* (adapted from Berberof *et al.* 2001; Felu *et al.* 2007). (Lower) Diagram of the typical domains of a *VSG* and the approximate location of the *C*-terminal deletion in TgsGP (adapted from (Gibson *et al.* 2010)).

In an effort to discover the factor that confers resistance to TLF-2 and APOL1, the genomes of both *T. b. brucei* and *T. b. gambiense* were interrogated (Berriman, 2005; Jackson *et al.* 2010). To date, only one gene has been found to be unique to *T. b. gambiense*, the gene encoding the *T. gambiense*-specific glycoprotein (TgsGP) (Berberof *et al.* 2001). This gene was found while attempting to discover truncated *VSGs* similar to *SRA* in *T. b. gambiense.* Unlike *SRA, TgsGP* is not found in an expression site but is rather transcribed from a core chromosomal locus. This region was formed by an inversion event that disrupted part of chromosome 2 that allowed an ordinarily silent region containing *VSG*s to be transcribed (Fig. 3) (Berberof *et al.* 2001; Felu *et al.* 2007). This inversion event is ancestral and specific to *T. b. gambiense* (Felu *et al.* 2007). A *VSG* or *VSG* pseudogene close to an AUT1 fragment cleaved by the inversion has since evolved into the *TgsGP* gene. *TgsGP* appears to be highly conserved across several *T. b. gambiense* disease foci so is likely to have evolved only once (Gibson *et al.* 2010). It differs from other VSGs in that it does not possess the conserved *VSG* C-terminal domain and possesses a GPI anchor related to that of ESAG6s (Fig. 3) (Felu *et al.* 2007). It is closely related to the *VSG* Tb10.v4·0178, sharing more than 80% genetic similarity, indicating a possible ancestral VSG (Gibson *et al.* 2010). Initially *TgsGP* was thought unlikely to be involved in human serum resistance as ectopic expression of the gene in *T. b. brucei* did not confer resistance (Berberof *et al.* 2001). However, this does not take into account other factors that may be present in *T. b. gambiense* and absent in *T. b. brucei* that are necessary for TgsGP to function. When the *TgsGP* gene was deleted from the genome of *T. b. gambiense*, parasites became sensitive to both human serum and APOL1 (Capewell *et al.* 2013; Uzureau *et al.* 2013), although the parasites were still resistant to TLF-1. Removal of the *TgsGP* gene from *T. b. gambiense* parasites ectopically expressing a functional *T. b. brucei* HpHbR resulted in the parasites becoming sensitive to TLF-1 (Capewell *et al.* 2013; Uzureau *et al.* 2013). How TgsGP confers resistance is still unknown as there is no direct interaction between APOL1 and TgsGP despite both being found in similar endosomal compartments (Uzureau *et al.* 2013). Serial modification and ectopic expression of *TgsGP* indicated the presence of a specific Β-sheet structure within the protein that is essential for TgsGP to function. It was hypothesized that this Β-sheet structure interacts with and strengthens the parasite's internal membranes to allow them to resist the pore-forming activity of APOL1. Support for this hypothesis is based on the observation that recombinant TgsGP and derived peptides integrate with artificial lipid bilayers and can affect lateral flow of the parasite surface membrane (Uzureau *et al.* 2013). This suggests that membrane fluidity is different in *T. b. gambiense* (that contains TgsGP) compared with *T. b. brucei* and suggests a causal relationship with the human serum resistance phenotype. This is indeed a tempting hypothesis, although a direct relationship between the observation and human infectivity has not been shown. Recombinant TgsGP and related peptides have a negligible effect on human serum resistance when incubated with *T. b. brucei.* This fact, coupled to the observations that ectopic expression of *TgsGP* in *T. b. brucei* does not confer resistance and that *TgsGP* knockout *T. b. gambiense* still displays a markedly higher resistance to human serum than other *T. b. brucei*, suggests other factors may be present in *T. b. gambiense* that function in concert with TgsGP (Berberof *et al.* 2001; Capewell *et al.* 2011; Uzureau *et al.* 2013).

One possible candidate for involvement is the cysteine protease protein family. These enzymes are heavily involved in degradation of host proteins, with several found in the endosomal pathway and lysosome. It has been demonstrated that cysteine protease inhibitors affect the ability of *T. b. brucei* to resist lysis by human serum, perhaps by affecting degradation of the TLF particles or APOL1 itself (Bishop *et al.* 2001). General inhibition of cysteine peptidase in *T. b. gambiense* strains without TgsGP reduced their capacity to resist human serum and APOL1 to a level approximately equivalent to *T. b. brucei* (Uzureau *et al.* 2013). RNAi and knockouts of *ICP* (inhibitor of cysteine peptidase) cause a general increase in cysteine protease expression in *T. b. brucei* and also raise human serum resistance, suggesting that these proteases could be a factor (Uzureau *et al.* 2013). However, an experiment that over-expresses cysteine proteases in a *T. b. brucei* with ectopic *TgsGP* expression has not been undertaken. This would confirm if high cysteine protease activity is indeed the missing factor necessary for TgsGP to function in *T. b. brucei*. While higher expression of cysteine proteases in *T. b. gambiense* may be the effector that leads to greater human serum resistance, it was also noted that the pH of the endocytic compartment of *T. b. gambiense* is slightly lower than that of *T. b. brucei* (Uzureau *et al.* 2013). This may increase the degradation of APOL1 and elicit a protective effect. Several *T. brucei* cysteine proteases, particularly cathepsins, are more active at low pH (O'Brien *et al.* 2008). Again, this hypothesis has not been tested and is at present conjecture.

Taken together, it would appear that the resistance mechanism of *T. b. gambiense* involves several factors: (1) reduction of lytic particle uptake by reducing expression and binding affinity of the HpHb receptor, (2) expression of *TgsGP* possibly affecting membrane fluidity and resistance to APOL1 disruption and (3) increased amounts of cysteine proteases that aid degradation of APOL1.

## EVOLVING PRIMATE RESISTANCE TO *T. B. RHODESIENSE* AND *T. B. GAMBIENSE*

**Fig. 4.**
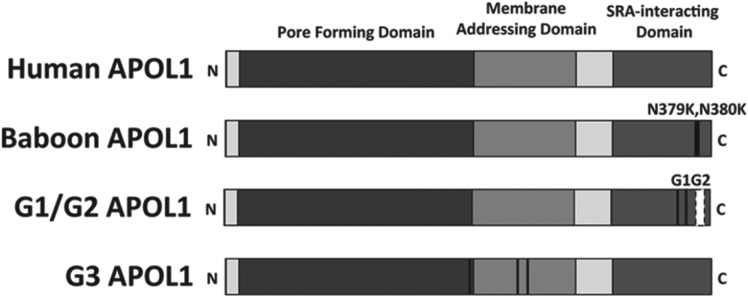
APOL1 variants. Several variants of APOL1 show differing lysis efficacy against trypanosomes. Wild-type human APOL1 can lyse *T. congolense, T. vivax* and *T. b. brucei*. Baboon APOL1 possesses two consecutive lysines rather than asparagines in the SRA-interacting domain of APOL1 (black lines). This reduces the binding affinity of SRA to APOL1, allowing the baboon protein to lyse *T. b. rhodesiense*. G1/G2 human alleles are under selection in populations of African descent and also possess modifications to the SRA-interacting domain of APOL1 (black lines, white box). Like baboon APOL1, these alleles demonstrate efficacy against *T. b. rhodesiense*. The G3 allele is under selection in populations exposed to *T. b. gambiense* but efficacy of this allele against trypanosome sub-species has not been assessed. Unlike G1/G2, G3 mutations lie within the pore-forming and membrane-addressing domains of APOL1 (black lines).

Although the mechanisms for how primates resist trypanosome infection and how some trypanosomes have overcome this resistance are becoming clearer, the evolutionary arms race between primates and trypanosomes continues. It is becoming apparent that mechanisms to combat infection of both *T. b. rhodesiense* and *T. b. gambiense* in primates have evolved and are continuing to evolve. Although trypanolytic APOL1 evolved in the ancestors of *catarrhine* primates, it appears to have diverged into two distinct lineages that exhibit differing efficacies of trypanosome lysis. APOL1 from the *hominidae* lineage (gorillas and humans) is effective against most trypanosomes (Seed and Sechelski, 1990; Lugli *et al.* 2004), except *T. b. rhodesiense* and *T. b. gambiense* while APOL1 from the *cercopithecidae* lineage (baboons and macaques) is also able to lyse *T. b. rhodesiense* (Seed and Sechelski, 1990; Kageruka *et al.* 1991; Thomson *et al.* 2009). *Cercopithecidae* lineage APOL1 is able to lyse *T. b. rhodesiense* due to a modification of the SRA interacting domain of APOL1 at the C-terminus of the protein (Fig. 4), which disrupts SRA binding after APOL1 has undergone conformational change (Lecordier *et al.* 2009; Thomson *et al.* 2009). Recombinant protein created by deleting this domain from human APOL1 is also able to lyse *T. b. rhodesiense*, suggesting that evolving a modification to this region of APOL1 would render humans resistant to *T. b. rhodesiense* (Lecordier *et al.* 2009). There is preliminary evidence that this may have occurred with the identification of selection for two specific APOL1 alleles (G1 and G2) in African Americans with ancestry in sub-Saharan Africa (Genovese *et al.* 2010). Both the G1 and G2 alleles of *APOL1* show modification of the SRA binding region (Fig. 4) and recombinant versions of these alleles, particularly G2, are able to reduce the growth of *T. b. rhodesiense* parasites *in vitro* (Genovese *et al.* 2010). However, presence of these alleles also correlates with increased incidence of kidney disease, particularly in individuals homozygous for either allele (Freedman *et al.* 2010; Genovese *et al.* 2010; Tzur *et al.* 2010). The maintenance of a trait that is beneficial to heterozygotes but deleterious to homozygotes parallels the well-described co-evolutionary interplay between sickle-cell anaemia and *Plasmodium* (Williams *et al.* 2005). However, to date, no case-controlled study using natural populations has been undertaken to test the hypothesis that G1/G2 selection is due to pressure from trypanosomes. A study examining APOL1 selection across Africa found extremely low prevalence for G2, and G1 was only present in a population not exposed to *T. b. rhodesiense* (Ko *et al.* 2013). They did, however, find a much higher number of non-synonymous mutations in the SRA binding domain of APOL1 than would be expected by chance.

While less well studied than *T. b. rhodesiense*, it is also possible that there are primates or human populations that have evolved APOL1 alleles that protect against *T. b. gambiense.* The Bambuti people of the Mbomo region in the Democratic Republic of the Congo have long been believed to be less susceptible to African sleeping sickness (Frezil, 1983), although no controlled study has been performed to test this assumption or investigate their APOL1 alleles. It is also becoming increasingly apparent that infection by *T. b. gambiense* is not always fatal with the recent identification of asymptomatic and self-cure cases from Côte d'Ivoire (Ilboudo *et al.* 2012; Jamonneau *et al.* 2012). The APOL1 sequences from these individuals have not been assessed, although a recent study assessing APOL1 selection across Africa has identified an allele termed G3 that is under selection in the Fulani people of Cameroon, a region affected by *T. b. gambiense* African sleeping sickness (Ko *et al.* 2013). This particular allele differs from the G1/G2 variants that have a mutation in the SRA interacting domain and instead possesses a mutation in the membrane-addressing domain of APOL1 (Fig. 4). It is, as yet, unknown if this variant is protective against *T. b. gambiense*. Looking outside human populations and to the *Cercopithecidae* lineage of APOL1, the baboon species *Papio papio* appears to only be transiently infected by *T. b. gambiense.* This species range overlaps with that of *T. b. gambiense*, while the ranges of other *Papio* species that are not resistant to the disease do not overlap (Kageruka *et al.* 1991; Lecordier *et al.* 2009; Thomson *et al.* 2009). It is possible that this species of baboon has evolved an APOL1 variant that is able to lyse *T. b. gambiense* due to exposure to the disease. Finally, while the majority of effort has primarily concentrated on finding APOL1 variants toxic to human infective trypanosomes, other proteins may also contribute. For example, specific alleles of IL-6 and IL-10 have been associated with resistance to *T. b. gambiense* in humans (Courtin *et al.* 2006, 2007; Garcia *et al.* 2006). A recent study has also reported evidence that alleles containing a duplication of the parasite receptor ligand, *HPR*, are present at high frequency in Central and West African populations endemic to *T. b. gambiense*, relative to the global distribution. However, whether such duplication offers any protective effect against *T. b. gambiense* sleeping sickness has not yet been experimentally tested (Hardwick *et al.* 2014). There was no significant link between *HPR* duplication and the presentation of sleeping sickness in children in affected areas, although this may be due to the low power of the study. To discover other possible factors involved in genetic susceptibility to African sleeping sickness, large-scale genome-wide association studies should be undertaken.

## DISCUSSION AND FUTURE DIRECTIONS

**Fig. 5.**
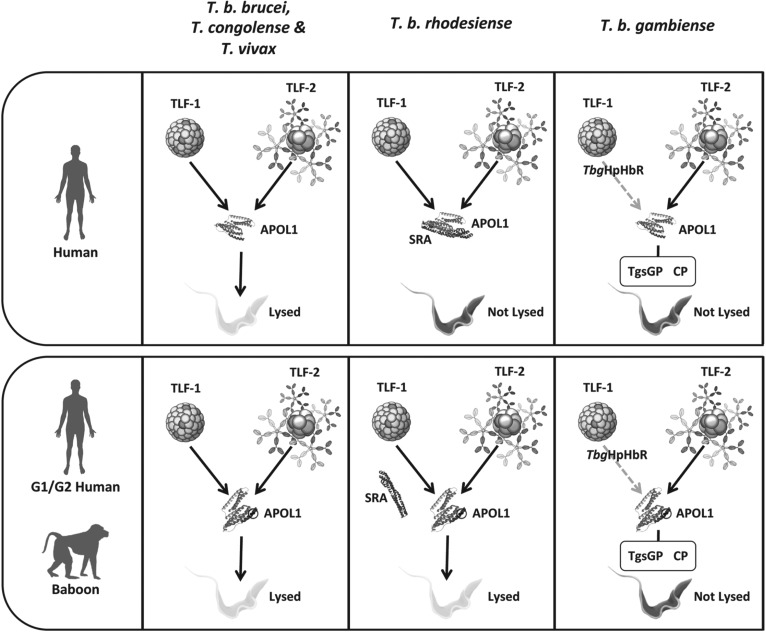
Summary of the co-evolutionary arms race between African trypanosomes and the primate host. Humans are protected from infection from the majority of Africa trypanosome species by two serum trypanolytic factors, TLF-1 and TLF-2, which exploit parasite nutrient scavenging pathways to take up and deliver lytic APOL1 protein to the lysosome. A conformational change in the low pH environment of the lysosome releases APOL1 and exposes domains that allow it to form anionic pores in the membrane, leading to osmotic imbalance and cell lysis. Two sub-species of *T. brucei* have evolved specific mechanisms to overcome this innate resistance. In *T. b. rhodesiense*, expression of the *SRA* gene, a truncated VSG, confers resistance to lysis by both TLF-1 and 2. Deletion of the VSG surface loops results, ultimately, in its trafficking through the endocytic pathway, where it is able to bind APOL1 and prevent pore-forming activity in the lysosome. In contrast, *T. b. gambiense* has evolved a complex, multi-component mechanism of HSR involving reduction in the binding affinity of *Tbg*HpHbR for TLF-1, the expression of a *T. b. gambiense* specific truncated VSG (*TgsGP*) which conceivably increases resistance of the lysosomal membrane to APOL1 disruption and enhanced expression or activity of cysteine proteases (CP) that aid degradation of APOL1 within the endocytic pathway. Recently, the discovery of certain APOL1 variants has suggested the evolution of counter measures to at least one of these resistance mechanisms. APOL1 from a subset of primates of the *cercopithecidae* lineage are naturally resistant to *T. b. rhodesiense* but not *T. b. gambiense* infection as a result of mutations in the SRA interacting domain that impair SRA binding and restore APOL1 activity. In an example of convergent evolution, two haplotypes displaying similar mutations, termed G1 and G2, have been identified in a number of human populations of African origin.

The co-evolutionary arms race between trypanosomes and primates is a fascinating case study in parasite and host interactions. As the mammalian adaptive immune system is rendered ineffective by the trypanosomes’ antigenic variation strategy, primates have evolved specific countermeasures to target trypanosomes utilizing the trypanolytic protein APOL1 bound to HDL (Summary: Fig. 5). By evolving a trypanolytic HDL, primates were able to exploit both the trypanosomes’ high lipid requirement necessary for fast turnover of the VSG coat to maintain antigenic variation and the lack of a haem production pathway in the parasite. However, two sub-species of *T. brucei* have evolved their own counters to overcome lysis by human serum and APOL1. Both resistance mechanisms appear to utilize a truncated *VSG* as an essential component, *SRA* in *T. b. rhodesiense* and *TgsGP* in *T. b. gambiense.* This highlights the evolutionary potential that the huge VSG repertoire provides this parasite. Although not covered during this review, there are also two other potential serum resistance mechanisms in *T. brucei* that do not utilize SRA or TgsGP. In East sub-Saharan Africa, a small number of human infective trypanosomes have been identified from *T. b rhodesiense* foci that do not possess the *SRA* gene (De Greef *et al.* 1989; Rifkin *et al.* 1994; Enyaru *et al.* 2006). In Western sub-Saharan Africa, sympatric with *T. b gambiense*, a further group of human infective trypanosomes has been identified. These are termed group 2 *T. b. gambiense* as they do not fit into the classical genetic profiles associated with the much more prevalent group 1 *T. b. gambiense* (Gibson, 1986). Unlike group 1, group 2 *T. b. gambiense* do not possess *TgsGP* and the resistance mechanism is variably expressed in a manner reminiscent of *T. b. rhodesiense*. The resistance mechanism does not appear to correlate with changes in expression site, however (Capewell *et al.* 2011). How non-SRA *T. b. rhodesiense* and group 2 *T. b. gambiense* are able to overcome APOL1 remains unclear, although it highlights the high potential for *T. brucei* to quickly evolve to infect new hosts and overcome innate immunity.

It is becoming apparent that the evolutionary arms race between primates and *T. brucei* is continuing, with several human and primate populations displaying resistance to both *T. b. rhodesiense* and *T. b gambiense*. Understanding the complex co-evolution that has occurred between trypanosomes and primates may lead to prospective disease interventions. For example, inhibitors or antibodies that target the essential proteins involved in each sub-species’ resistance mechanism (SRA and TgsGP) would render these parasites susceptible to normal innate resistance and APOL1. An alternative approach is to identify APOL1 variants or other genes present in trypanosome-resistant human and primate populations that may serve as a universal therapy for all African trypanosomes. Once identified, these APOL1 variants must be targeted to trypanosomes as APOL1 uptake when not bound to a facilitating ligand is slow and inefficient (Vanhamme *et al.* 2003; Baral *et al.* 2006). One proposed solution involves the distribution of transgenic cows across Africa that express either recombinant baboon APOL1 (Thomson *et al.* 2009) or truncated human APOL1 (Lecordier *et al.* 2009). Mice models expressing these genes ectopically are unable to be infected by either *T. b. brucei* or *T. b. rhodesiense*, if expressed in concert with HPR and APOA1 (Thomson *et al.* 2009). An alternative proposed delivery mechanism is the conjugation of variant APOL1 to antibody fragments that target conserved motifs on parasite VSGs (Baral *et al.* 2006). A caveat to these proposed interventions is that the APOL1 variant used by each is not lytic to the most prevalent human infective trypanosome, *T. b. gambiense* (Lecordier *et al.* 2009; Thomson *et al.* 2009). It is possible that usage of such APOL1 variants would select for increased incidence of human disease by eradicating the competitor species such as *Trypanosoma congolense, Trypanosoma vivax* or *Trypanosoma brucei*. Nevertheless, it is clear that understanding the co-evolution of primates and African trypanosomes is a powerful tool in combating human trypanosomiasis.
